# Case of Fractured Rib, with an External Wound

**Published:** 1826-09

**Authors:** 

**Affiliations:** the Middlesex Hospital


III. *Case of fractured Rib, with an external Wound.* Treated at the Middlesex Hospital, by Mr. Shaw.

William Yateman, a bricklayer, aged twenty-five, was brought to the hospital on the 10th of June, 1826. He stated that he had fallen from a ladder, and struck on a spike of the railing beneath him. There was a wound near the inferior margin of the right scapula, just below the axillary cavity and the inferior edge of the latissimus dorsi: it was of a size sufficient to admit with ease the fore-finger, which was readily passed forwards, and was then found to be closely in contact with the axillary artery. The third rib was discovered to be fractured near its junction with the cartilage. On introducing the finger in the opposite direction, it passed behind the scapula. No air escaped from the wound, nor was there any emphysema. The respiration was difficult, and attended with pain; the pulse ninety-six, full and strong.

Eighteen ounces of blood were taken from the arm, which produced syncope. The edges of the wound were brought together with adhesive straps, and a bandage was put around the chest.—The bowels were well opened; and he took Saline Medicines, with Tartarised Antimony, in nauseating doses, three times a-day.

Next morning (11th), the breathing was not much relieved; pulse ninety-two, full and strong; tongue covered with a brownish fur; bowels confined.

He was bled till faintness came on, and purged with Senna and Salts.

In the afternoon, the pulse having again risen, the bleeding was repeated; and in the evening, as he complained of some pain in the left side, twelve leeches were applied, which gave him great relief.

He was likewise ordered to take, Opium gr. ¼; Antimon. Tartar. gr. ¼; Calomel gr. ij. every six hours.

On the 13th, he complained of pain in the chest and cough; and was again bled until syncope was produced.

On the 15th, the wound had begun to suppurate, and discharged a small slough. He was somewhat better; but the pulse still continued full and strong, and the cough distressing.

The bleeding was repeated; and the Calomel, Antimony, and Opium were given at night only; while a Demulcent Draught, containing Compound Tincture of Camphor, was administered three times a-day.

Next day, the cough and pain in the chest, although less, were still troublesome; and his pulse, though not so strong, was still full.—The bleeding was again repeated.

From this period he improved. The wound long continued to discharge, but was completely cicatrised by the 7th of July, when he was allowed to go out. Having committed some excess, his cough returned on the 9th: this, however, was quickly removed by a blister on the chest; and he was discharged, perfectly recovered, on the 18th of July.

It is unnecessary to add, that this man owed his recovery entirely to the active antiphlogistic means employed; having lost above eighty ounces of blood in the course of a few days.

---

